# Aspects of Prophylactic Vaccination against Cervical Cancer and Other Human Papillomavirus-Related Cancers in Developing Countries

**DOI:** 10.1155/2011/675858

**Published:** 2011-07-19

**Authors:** Kari Natunen, Johannes Lehtinen, Proscovia Namujju, John Sellors, Matti Lehtinen

**Affiliations:** ^1^School of Health Sciences, University of Tampere, 33014 Tampere, Finland; ^2^Faculty of Social Sciences, McMaster University, Hamilton, ON, Canada L85 4L8

## Abstract

Cervical cancer and other human papillomavirus- (HPV-) related cancers are preventable, but preventive measures implemented in developing countries and especially in low-income rural regions have not been effective. Cervical cancer burden derived from sexually transmitted HPV infections is the heaviest in developing countries, and a dramatic increase in the number of cervical cancer cases is predicted, if no intervention is implemented in the near future. HPV vaccines offer an efficient way to prevent related cancers. Recently implemented school-based HPV vaccination demonstration programmes can help tackle the challenges linked with vaccine coverage, and access to vaccination and health services, but prevention strategies need to be modified according to regional characteristics. In urban regions WHO-recommended vaccination strategies might be enough to significantly reduce HPV-related disease burden, but in the rural regions additional vaccination strategies, vaccinating both sexes rather than only females when school attendance is the highest and applying a two-dose regime, need to be considered. From the point of view of both public health and ethics identification of the most effective prevention strategies is pivotal, especially when access to health services is limited. Considering cost-effectiveness versus justice further research on optional vaccination strategies is warranted.

## 1. Background

### 1.1. Human Papillomavirus and Cervical Cancer

Human papillomavirus (HPV) is the main cause of cervical cancer [1], the third most common cancer in women [2]. There is, however, growing evidence linking genital HPV infection to other anogenital cancers (anus, vulva, vagina, and penis) and head and neck cancers in both men and women [3–11]. HPV is carried by both females and males and can spread with high (up to 0,6 per act for HPV16) transmission probability [12], and most (up to 70–80%) people will get infected during their lifetime [13]. Thus, HPV can be characterised as a ubiquitous, sexually transmitted infection (STI) causing significant disease burden in both sexes, but especially in women with up to 6-7% lifetime risk of developing cervical cancer in Latin America [14].

Of the estimated 530000 annual cases of cervical cancer, 86% occur in developing countries [2]. Its standardised incidence ratio (world, 100) is substantially higher in the developing countries (116) than in the developed countries (60). It is important to note that cervical cancer is the most common cancer in women in most parts of Africa, Central America, Southern Asia, and Melanesia. [3] It is also the most important cause of years of life lost in Latin America and the Caribbean, and among cancers in the populous regions of Sub-Saharan Africa and South-Central Asia [14]. Furthermore, largely due to changes in sexual risk taking behaviour and (in some countries) dynamic state of epidemics by high-risk (hr) HPV types [15, 16], the number of cervical cancer cases has been predicted to rapidly increase (up to 90%) by 2020 in developing countries if no intervention is implemented [17, 18].

### 1.2. Occurrence of Other HPV-Related Cancers

It has been established that the HPV attributable proportion in cancers of the anus, vagina, penis, vulva, oropharynx, and oral cavity is 25% or higher [8, 11, 19–21]. There are clear signs that the incidence of most HPV-related cancers is increasing. The incidence rates of anal cancer in Scotland and England have nearly doubled in both women and men from 1986 to 2003 [22]. Increase in the incidence of anal cancer is also reported in Australia [23]. In the developed countries, also the incidence of HPV-related head and neck cancer is rapidly increasing especially in males and in the younger birth cohorts [5, 6, 9–11, 24]. In Australia, Netherlands, Sweden, and USA, the incidence of HPV-related tonsillar cancer has rapidly increased during recent years [7, 23, 25, 26]. Similar trends have not yet been reported for the developing countries.

### 1.3. Occurrence of Infections with High-Risk HPV Types

Most of the genital infections with hrHPV type(s) are asymptomatic and heal without treatment. The risk of cervical cancer increases as the hrHPV infection persists. Over 70% of cervical cancers are attributed to HPV types 16 and 18 and approximately 20% to HPV types 31, 33, 35, 45, 52, and 58 [3, 27]. Precancerous cervical lesions usually appear within 5 years in individuals with an established persistent infection with HPV types 16 and 18. A majority of other HPV-related cancers are also attributable to HPV types 16 and 18. For example, it is estimated that 24% of cancers in the mouth are associated with HPV and 95% of these cancers are attributable to HPV types 16 and 18. Over 80% of anal cancers are associated with HPV and 92% of these cancers are attributable to HPV types 16 and 18. Thus, it can be assumed that targeting HPV types 16 and 18 and a remarkable proportion of phylogenetically related hrHPV types (31, 33, 45) by prophylactic vaccination [28–30] would play a significant role in preventing all HPV-related cancers [27]. 

Prevention of hrHPV infections could decrease the incidence of numerous cancers in both sexes [5, 31]. In healthy men, HPV infection in the genital tract is very common (from 35% to 73%) [32]. Alike other STIs, HPV transmits more easily from men to women than from women to men [3]. Male circumcision and use of condoms prevent the spreading of hrHPV infections, which probably explains the low incidence of cervical cancer in countries such as Israel where circumcision is widespread [3, 33]. Racial differences have been reported showing that African-Americans are less likely to have HPV-positive head and neck (oropharyngeal) cancers [34, 35]. Reasons for the recent increase of oropharyngeal HPV-related cancers, especially in younger male birth cohorts in the developed countries, are not clear, but changes in sexual behaviour, efficiency of HPV transmission through oral sex, and lack of protective immunity from hrHPV infections of the genital mucosae have been suggested [6, 36]. 

Various studies indicate that a high number of lifetime sexual partners, tobacco smoking, parity, oral contraceptive use, and coinfections with *Chlamydia trachomatis* and HIV increase the risk of acquisition of hrHPV infection [37–42]. The impact of cofactors on the acquisition of infections with multiple hrHPV types has not been studied largely [43, 44].

STIs are a serious health problem in developing countries, and several studies indicate that conventional STIs increase the likelihood of HIV transmission [45]. HPV infections in both females and males are also risk factors for HIV acquisition [46–48]. Prevention of HPV infections could play a part in preventing other STIs as well [49]. It is also highly likely that HPV-related neoplasia progresses faster in HIV-positive people [50].

## 2. Prevention of HPV-Related Cancers

### 2.1. HPV-Related Cancers and Cancer Mortality Are Preventable

Cervical cancer is a disease which can be prevented. Similar to liver cancer that is secondary to hepatitis B infection, cervical cancer has a recognized, single necessary cause, HPV. Application of cervical cytology in population-based screening programmes has significantly lowered the incidence of cervical cancer in developed countries [3]. Cervical cancer incidence and mortality decreased markedly in the Nordic countries, Europe, Canada, and USA due to the implementation of cervical cytology in health care, most notably in population-based screening programmes [51–54]. Mortality from cervical cancer has also substantially declined since the 1960s in Europe, but there are still large country-specific differences [55]. Cervical cancer mortality is substantially higher in Eastern Europe than in other parts of Europe. Increases in the incidence of and mortality from cervical cancer have been reported in the last 15 years resulting from HPV epidemics and a drop in the number of women participating in the screening programmes [55]. 

Mortality rates of cervical cancer are lower than incidence rates with a ratio of mortality to incidence of 55% [3]. Survival rates are, however, lower in the developing countries [56–58], and the differences in the ratios of mortality to incidence between developing and developed countries are significant. The ratios range from 20% in Switzerland to 80% in most African countries. Latin America and South Asia have ratios of 40–55% [3]. The differences can be explained by the stage at which cancer is detected, access to health services, and adequacy of treatment.

### 2.2. New Means for Primary Prevention of HPV-Related Cancer

Currently there are two licensed prophylactic HPV vaccines, a bivalent vaccine (Cervarix) against HPV types 16 and 18 and a quadrivalent (Gardasil) vaccine against HPV types 6, 11, 16, and 18 available (FDA 2006; EMEA 2007). In order to be prophylactic, both vaccines need to be administered before the individual is exposed to HPV types covered by the vaccine. According to reports from the major phase III trials, the vaccines prevent from 97 to 98% of infections caused by HPV types 16 and 18 [59, 60]. Both vaccines have shown a significant cross-protection also against HPV types 31 and 45 [28, 29]. The bivalent vaccine has also shown cross-protection against HPV types 33 and 51 [30]. As indicated above, over 70% of cervical cancers are attributed to HPV types 16 and 18, and approximately 20% to HPV types 31, 33, 35, 45, 52, and 58 [3, 27] which fits the cross-protection efficacy and reported 87% overall vaccine efficacy against CIN3+ [29]. It is assumed that both HPV vaccines, in preventing hrHPV infections, prevent other HPV-related cancers besides cervical cancer with high to moderate efficacy [61].

It is known that the existing vaccines are most efficient for antibody production when administered to early adolescents. Both males and females had higher antibody responses at the age of 9–15 compared to the age of 16–26 [62, 63]. There is, however, no concrete information available on long-term (>10 years) efficacy of the vaccines and necessity for a booster. One model has predicted an over 20-year protection but at the moment the predictions rely only on assumptions [64]. It is not known if HIV infection will affect the efficacy of HPV vaccines [65], but smoking does not seem to affect HPV vaccine-induced antibody response [42]. Booster doses work very well and produce higher antibody levels when measured one month later [62]. HIV positivity as such does not hamper development of HPV antibodies following natural infection [66], whereas smoking does [42]. Type replacement, that is, how nontargeted hrHPV types that may have competitive advantage [67] will behave following mass vaccination, is an open question, but the likelihood of the kind of type replacement seen following bacterial vaccination is small due to the different biology of viral and bacterial infections [68, 69].

## 3. Primary Prevention Strategies in Developed Countries

According to WHO, 22 countries in low-resource regions have included an HPV vaccine in their vaccine programmes [70]. Adopted vaccination strategies include offering the vaccine only to 12–15-year-old girls or only to a certain proportion of the female population of the same age. The vaccination strategies are in line with the results of numerous cost-effectiveness studies which suggest that with high vaccine coverage (≥75%) vaccination of males would not be cost effective [71–82]. Vaccinating males becomes cost effective by assuming a low to moderate (30%–50%) coverage in females [74].

The assumptions behind these recommendations and the superior cost-effectiveness of female vaccination strategy are that the health service system covers all the regions equitably and that adolescent girls have both access to the health services and are willing to use it. However, the probability of preventing other HPV-related cancers both in females and males has not been taken into account. Emerging information on HPV-related cancers in men may change the conclusions of cost-effectiveness modelling. In Australia, it is estimated that one quarter of the preventable cancers are in men [23], but this most likely varies between countries. 

There is not enough information on how vaccinating males would change the transmission of HPV, but it is possible that vaccinating only females could result in an increase in HPV transmission like in the case of Rubella vaccination and Rubella acquisition by young adult females in the UK [83]. British data also suggests that due to sexual behaviour characteristics British men are at greater risk of being exposed to, contracting, and transmitting HPV infection than females. Each vaccinated male would therefore reduce the infection risk more than a vaccinated female [84]. It is important to estimate if this assumption is true, and the extent of the possible difference. Overall, the highly infectious nature (high transmission probability) of HPV supports the idea of vaccinating both sexes. It is also possible that vaccinating the same number of males and females as in the female-only vaccination strategy would decrease the prevalence of hrHPV infections slightly in a steady state of hrHPV epidemics [85] and in dynamic state of the hrHPV epidemics (as is the case in many countries), and it is likely that the impact of male vaccination would be higher.

The questions concerning HPV vaccine efficacy on males and the possible effect of vaccination on HPV transmission are valid as it is known that the vaccine against herpes simplex virus type 2 is not effective on males [86] but it could still have a profound effect on HSV-2 occurrence through herd immunity provided viral shedding is significantly reduced [87]. Prevention of infectious diseases comparable to HPV with vaccines is based on producing herd immunity through a sufficient coverage in susceptible individuals to reduce transmission [87]. The quadrivalent HPV vaccine is proven efficacious in males, likely prevents HPV transmission [88], and has been shown to reduce HPV 6/11-associated disease burden. Vaccinating males is currently not recommended by the WHO, but the impact of herd immunity on female cancers and other HPV-related cancers may need to be reconsidered [89, 90].

High vaccine coverage is needed to produce herd immunity, and the key question is whether sufficient coverage can be achieved by vaccinating only females. Sufficient coverage has been achieved only in the UK (75%) and Australia (70%). In the Netherlands (50%), Germany (40%), and USA (25%), the coverage is neither enough to protect significant proportions of females nor to produce herd immunity. Protection is effective on an individual level, but in the absence of herd immunity the unvaccinated remain unprotected. At present, the questions concerning coverage in both sexes and the magnitude of herd immunity remain open [65, 87, 90]. Mathematical models suggest that vaccinating both males and females could produce herd immunity and an impact both on hrHPV prevalence and occurrence of cervical cancer with considerably lower coverage than vaccinating females only [12, 89, 90]. This could be a decisive factor in the low-income areas where there are problems with access to health services. Other reports indicate better results (reduction of HPV prevalence in unvaccinated females by 86–96% versus 7–31%) with a vaccine coverage of 80% in females and males versus 80% in females only [91], but there is no evidence-based data available yet. 

## 4. Prevention Strategies of HPV-Related Cancers in the Developing Countries

### 4.1. A Different Point of View

The need for cervical cancer prevention is the greatest in developing countries where the burden of cervical cancer and other HPV-associated cancers is the heaviest, and preventive measures have not been/cannot be implemented consistently. From the point of view of global justice, the prevention of cervical cancer should be a priority in countries where its burden is the heaviest. The Global Alliance for Vaccines and Immunisation (GAVI) considers HPV vaccines among the vaccines that would have the biggest impact on the disease burden in developing countries. Within the developing countries (and in some developed or middle-income countries), the situation in rural regions with major problems of access to health services poses various questions concerning equity and justice [92]. In the reality of overall scarce resources, rural regions tend to suffer the most [93]. This is exemplified in how screening has failed to make an impact in developing countries and especially in rural regions with problems of access to all cervical cancer prevention health services, including screening, diagnosis, treatment, and followup [53, 94]. The poor (<50%) acceptance of cervical screening by a significant proportion of females in the younger birth cohorts has resulted in a comparable loss of impact with consequent increase in cervical cancer incidence in the developed countries. In the developing countries the accessibility of health services is far too low to guarantee desired impact overall. HPV vaccines offer qualitatively different (primary/complete versus secondary/incomplete prevention) possibilities for preventing cervical cancer in women and other HPV-related cancers in both women and men. 

Due to the assortative nature of common sexually transmitted infections like HPV, the sufficient coverage to produce herd immunity effect is relatively low [95]. In the case of HPV, with a narrow window of applicability (before sexual debut), it is advisable to decide early enough if the vaccine is offered only to females or to both sexes, that is, all potential carriers of HPV infection. The question is pertinent in all regions where achieving (access to or acceptability of an HPV vaccine) high vaccine coverage is challenging. The challenges are numerous varying from financial restraints of access to cultural or religious acceptability of the vaccine.

In addition to access to health services (in this case vaccination or screening), it should be noted that the success of a vaccination or a screening programme is partly dependent on decision making by the objects of the intervention or other parties like parents, spouses, other family members, influential persons in the community, and politicians [96]. In the case of HPV, attitudes towards vaccination are more positive in persons who have more information on the vaccine, HPV, and the causality between HPV and cancer [97]. It is probable that those who have the least information are less likely to participate than those with solid/improved information. The average, complete nonacceptability does not exceed 10–15% [96–106] but can be further reduced with health education. According to numerous studies, women and men in general are unaware of the causality between HPV and cervical cancer, and it can be assumed that the connection between HPV and HPV-related cancers is even less generally known. Attitudes towards HPV vaccination are, however, generally positive [106] which is probably due to positive attitudes towards vaccinations in general [107]. 

HPV is such a common infection that a risk-group intervention alone is not likely to produce good results and have an impact. A high-risk group strategy based on behavioural characteristics would also be problematic to implement for a variety of reasons, most notably the inability to identify people at risk. Moreover, HPV does not have a tight core group as many classical STIs [108]. A useful strategy here might be to implement “add-ons” for risk groups in addition to a general vaccination programme. In the case of HPV, there may be two different risk groups: those who are at risk of infection because of risk-taking behaviour and those who are at risk of nondetection of sequelae because of geographical differences in access, that is, rural versus urban residence. Due to the ubiquitous nature of HPV infection it is reasonable that the WHO recommendation takes only the latter risk group into account by recommending starting a phased introduction in populations who do not have access to screening [109].

Cervical cancer is associated with poverty on both the global [3] and regional scalen [110–112]. The same association can be seen in cancer incidence and mortality in general [113, 114]. Poverty is the most common source of inequity along with gender, ethnicity, religion, geography, age, education, and social status [115–118]. It has been argued that health inequity in the developing countries is likely to increase if HPV vaccination programmes are not implemented [92, 119]. Cervical cancer mortality rate, which can indicate the effectiveness of screening, diagnosis, treatment, and followup, is proportionally higher (up to 18 times) in rural than in urban areas [110–112, 120]. People with low social status living in the developing (or middle-income) countries and in the rural regions are the least well off in the case of HPV-related diseases. This again highly favours HPV vaccination as a prevention strategy in low-income areas with special emphasis on regional characteristics. 

This regional aspect has its implications in terms of judging different methods and optional preventive strategies that can guarantee access to and coverage of primary prevention. In the urban areas the recommended prevention strategies might be efficient to significantly reduce HPV disease burden because there are fewer problems connected to access and coverage. The challenges of implementing HPV vaccination programmes in practice are similar to some elements of implementing screening programmes, that is, financial constraints, competing health needs, and limited human resources. The infrastructure and logistical capacity needed are much more limited in rural regions and will require investments.

### 4.2. Optional Strategies for Rural and Urban Regions

The question is whether we can opt for a single strategy for a country or a group of countries or we should look at regional characteristics linked to access in deciding which strategy to adopt case by case. Malmqvist et al. [121] have stated that strategies aiming at herd immunity (providing the vaccine to males and females) might be the best way to prevent cervical cancer from the point of view of justice. Strategies aiming at herd immunity in the developed countries would also protect those who do not have access to the vaccine (or screening) or who do not accept vaccination or screening. Strategies that are doable in developed countries may not be practical in low-resource regions. Thus, it is appealing to contemplate different strategies for rural and urban regions taking into account access/distance to health services and opting for regional and/or population subgroup-specific strategies in order to achieve the highest possible impact. 

In case mass vaccination would be offered, several strategies exist. A few relevant regional or even community-level strategies from the point of view of developing countries and/or regions are described as follows:

1. vaccinating females at the age of 12–15:
  - unrealistic targeting a coverage of 70%,
  - questionable herd immunity effect,
  - marginalized females excluded;

(2) vaccinating females when school attendance is at the highest (10–12 years of age):
  - unrealistic targeting a coverage of >80%,
  - prioritizing vaccinating younger girls before school attendance drops—need for boosters?
  - questionable herd immunity effect,
  - marginalized females excluded;

(3) vaccinating females and males at the age of 12–15:
  - targeting a coverage of >40% in all regions,
  - cost effectiveness,
  - acceptance of the vaccine for boys?
  - marginalization tackled by the herd immunity effect?

(4) vaccinating females and males when school attendance is at the highest (10–12 years of age):
  - targeting a coverage of >50% in all regions,
  - acceptance of the vaccine for boys?
  - marginalization tackled by the herd-immunity effect.

All strategies would be school based with community outreach activities in regions where school attendance is low and equally there would be a need for information campaigns to adolescents, service providers, decision makers, schools, and parents [122]. Information campaigns for both sexes with a notion to all HPV-related cancers might further increase the acceptance of HPV vaccine. It would also be important to address local or cultural issues which are linked to vaccines [93].

In terms of HPV vaccine coverage in developed countries, the highest rates have been achieved by school-based vaccination programmes [123]. School-based demonstration projects have shown promise in terms of coverage and compliance. In a universal school-based vaccination programm the adolescents who go to school may bring also nonattendees to the vaccination site [124]. School attendance of girls has increased in developing countries from 78% in 1990 to 85% in 2005 [125, 126]. Rates of primary school completion follow closely the enrolment figures [127]. The gender gap between boys and girls has disappeared in East Asia, Latin America, and Eastern and Southern Africa and is diminishing both in urban and rural regions and within economic quintiles [125, 127].

The challenges of a school-based programme are many. School-based programmes may not be feasible if sufficient resources are not allocated to providers [128]. School attendance in girls during adolescence may be lower than is needed for effective coverage, and certain high-risk groups might not be reached at all [124, 129]. 

The project funded by PATH, an American NGO, in Peru, Uganda, India, and Vietnam reached coverage of 80–95% in 9–14-year-old girls in selected schools demonstrating a high acceptability of the HPV vaccine [130]. The PATH project is also producing vital practical information on introducing HPV vaccines in developing countries (e.g., in certain cultures parents do not have documentation concerning date of birth, there may be undue concerns for fertility, emphasis on cancer prevention, etc.). Even though HPV vaccination shares many barriers with cervical screening [131], it seems that barriers linked to acceptation of HPV vaccine might be easier to deal with. For those females who agreed to be vaccinated, completion of the three-dose regimen was over 90% in Peru [130]. It is not clear whether vaccination rates such as these would be achievable in a nonresearch setting.

Optional preventive strategies, regional strategies, or mixed strategies, offering vaccination only to girls in some wealthier regions, and to both sexes in certain low-income regions could tackle the problems linked with coverage and access. In some areas, it might be more feasible to have boys vaccinated because of higher school attendance, and this could lessen problems of coverage even though fewer girls would be reached. Other preventive acts such as male circumcision [132] and use of condoms [133] make this a problem which has a solution that includes actions by both males and females. Tackling the problem as gender-free problem might promote vaccine acceptance and create political will [128]. Hence, the proposed “add-ons” would include vaccinating both sexes to achieve maximal coverage and acceptance. Targeting the early adolescents when school attendance is at the highest but before sexual debut might be a problem. In a study conducted in South Africa, some parents expressed their fear that vaccination at 11 years or older would already be too late [128].

The questions concerning dosage are closely linked with logistics, vaccine storage, vaccine acceptability, and cost effectiveness. The present vaccines are administered in three doses, but it is possible that a two-dose regimen could be enough to provide protection. This would have a significant effect mainly on costs but equally to vaccine acceptability and accessibility. In a study on Kenyan women, the acceptability of a three-dose vaccine regimen was only 31% compared to 86% for a one-dose regimen [105]. It is known that present vaccines do not provide effective protection in a one-dose regimen but a two-dose regimen remains possible. 

Further cost-effectiveness studies are needed taking into account other HPV-related cancers besides cervical cancer. Regional characteristics and problems linked with access and coverage should be addressed as well. Vaccination strategies including catch-up vaccination in older females or addressing early adolescents after the sexual debut should be linked with the most accessible screening and treatment methods such as the single visit approach (VIA and cryotherapy) to ensure that the means of prevention would protect those who may be already HPV infected and for whom the prophylactic vaccine cannot be effective.

Information about HPV vaccination and HPV-related cancers continues to emerge, but more research is needed especially on the long-term impact of vaccination, duration of protection, male vaccination, and reduction of HPV transmission. The GAVI Alliance subsidises the provision of vaccines to the poorest countries and is currently reviewing HPV vaccine as a candidate for sustainable financing. Even with secured financing, there is no simple answer concerning which strategy should be adopted in the developing countries.

## 5. Conclusion

HPV vaccination is the most promising way to prevent cervical and other HPV-related cancers in developing countries. The vaccine is effective, safe, and widely accepted. Vaccination strategies have an important effect on the success of any vaccination programme. In the case of HPV, it is crucial to reach at least 70% of females or 40–50% of both sexes before sexual debut. Currently, only the option of vaccinating females is recommended by WHO.

Optional preventive strategies, regional strategies, or mixed strategies in rural low-income regions could solve the problems linked with HPV vaccination coverage, access, and acceptability. Regional characteristics affect fundamentally the feasibility of HPV vaccination strategies as it has already been proven with screening. Differences between regions in terms of access to health services increase the need to adopt region-specific HPV vaccination strategies that are not currently deemed as cost effective. Emerging information on HPV-related cancers in both women and men and the feasibility of achieving high vaccine coverage in rural regions might produce different results in terms of cost effectiveness, and this might result as a change in strategic aims and recommendations. 

Targeting rural low-resource regions with specific vaccination strategies should be a priority from the point of view of ethics and public health. The number of cervical cancer cases is estimated to increase dramatically in developing countries if no intervention is implemented, and the trend is probably even stronger in regions where access to health services is limited. Whatever option is chosen, it is vital to merge any vaccination strategy with appropriate screening methods and sexual education.
